# Experimental Study on Shear Behavior of Steel Fiber Reinforced Concrete Beams with High-Strength Reinforcement

**DOI:** 10.3390/ma11091682

**Published:** 2018-09-11

**Authors:** Jun Zhao, Jingchao Liang, Liusheng Chu, Fuqiang Shen

**Affiliations:** 1 School of Civil Engineering, Zhengzhou University, Zhengzhou 450001, China; zhaoj@zzu.edu.cn (J.Z.); cls981@163.com (L.C.); 2 Graduate School of Engineering, Kobe University, Kobe 657-8501, Japan; shenfq_edu@163.com

**Keywords:** high-strength reinforcement, steel fiber, diagonal crack, failure modes, shear capacity

## Abstract

Many researchers have performed experimental and theoretical studies on the shear behavior of steel fiber reinforced concrete (SFRC) beams with conventional reinforcement; few studies involve the shear behavior of SFRC beams with high-strength reinforcement. In this paper, the shear test of eleven beams with high-strength reinforcement was carried out, including eight SFRC beams and three reinforced concrete (RC) beams. The load-deflection curve, concrete strain, stirrup strain, diagonal crack width, failure mode and shear bearing capacity of the beams were investigated. The test results show that steel fiber increases the stiffness, ultimate load and failure deformation of the beams, but the increase effect of steel fiber decreases with the increase of stirrup ratio. After the diagonal crack appears, steel fiber reduces the concrete strains of the diagonal section, stirrup strains and diagonal crack width. In addition, steel fiber reduces crack height and increases crack number. Finally, the experimental values of the shear capacities were compared with the values calculated by CECS38:2004 and ACI544.4R, and the equation of shear capacity in CECS38:2004 was modified to effectively predict the shear capacities of SFRC beams with high-strength reinforcement.

## 1. Introduction

With the increase of large-scale structures such as high-rise buildings and large-span bridges, the requirements for the strength and performance of materials have become higher and higher. For steel bars, the high-strength reinforcement with high strength, great corrosion resistance and good ductility is used to replace the conventional reinforcement in concrete structures, which can reduce the total amount of reinforcement and increase the spacing of reinforcement, thereby reducing construction difficulty and ensuring concrete pouring quality [1,2,3].

In recent years, many scholars have done large numbers of experimental studies on the shear behavior of beams with high-strength reinforcement. Munikrishma et al. [4,5] tested the shear behavior of RC beams with high-strength stirrups (yield strength of 690 MPa) and conventional stirrups (yield strength of 413 MPa). When the shear capacities of the beams were approximately equal, the required number of high-strength stirrups was less than that of conventional stirrups. Lee [6] tested the simple supported beams with different strength of concrete and stirrups (yield strengths of 334 MPa, 480 MPa, and 667 MPa, respectively). The results show that when the concrete strength of the beams is the same, the higher the yield strength of the stirrups, the greater the shear capacity. Sumper [7] compared the simple supported beams with high-strength reinforcement (yield strength of 827 MPa) and conventional reinforcement (yield strength of 427 MPa). The results indicate that the shear capacities of the test beams are improved by replacing conventional stirrups with high-strength stirrups, and when the longitudinal reinforcements are also used with high-strength steel, the shear capacity increases further.

Although the use of high-strength reinforcement can increase the shear capacities of RC beams, the high stress of high-strength steel bars at service stage will cause problems such as large crack width and deflection [6], which is not conducive to giving full play to the strength of high-strength reinforcement. Besides, because the ordinary concrete has large brittleness and low tensile strength, it is easy to cause cracking of RC beams with high-strength reinforcement. If steel fiber is added in concrete, the tensile strength [8,9], bending strength and flexural toughness of the concrete [10,11] can be improved, and the crack width and deflection of the beams with high-strength reinforcement can also be reduced [12]. At the same time, the advantages of high-strength reinforcement and steel fiber can be fully played.

Many experimental studies have verified that steel fiber can improve the shear performance of RC beams [13,14,15,16,17,18,19,20,21,22,23,24,25,26,27,28,29]. For example, adding adequate quantities of steel fiber into concrete can increase shear strength; due to the action of steel fiber in the tensile zone, the formation and development of the crack are delayed, and the tensile stiffness is enhanced to increase the integral stiffness; the spacing of the fibers is smaller than that of the stirrups, which means greater effectiveness in the crack-arresting mechanism and better distribution of tensile cracks, although the crack patterns of SFRC beam is similar to that of RC beam. Considering that the main effect of steel fiber is in the tensile zone, Gao and Zhao [17] tested the shear behavior of RC beams with different SFRC thickness and found that the shear capacity increased by 26–50%, while the crack width was reduced. In addition, the feasibility of steel fiber replacing stirrups was studied by some scholars. The contrast test of SFRC beams (stirrup spacing of 250 mm) and RC beams (stirrup spacing of 150 mm) was carried out by Ding [19], the results show that the shear capacities of SFRC beams are 14–18% higher than that of RC beams, and the steel fiber can replace a part of the stirrups. Based on a series of experiments, Narayanan and Darwish [20] conclude that steel fiber cannot completely replace stirrups when the structural elements are subjected to very high shear stress. However, the addition of steel fiber can reduce the brittleness of the failure, which can change the beam from brittle shear failure into ductile flexural failure. This conclusion is also found by other researchers [13,14,22,23,26,29].

Although there are many research achievements on shear behavior of SFRC beams, the shear behavior of SFRC beams with high-strength reinforcement are relatively few. At present, high strength reinforcement are not included in the design codes for SFRC structures of China and the United States. CECS38:2004 [30] and ACI544.4R-88 (Reapproved 2009) [31] were respectively compiled on the basis of GB50010-2002 [32] and ACI318-08 [33], in which the maximum yielding strength of steel rebar of GB50010-2002 and ACI318-08 are 400 MPa and 420 MPa, respectively. Therefore, it is necessary to further study the shear behavior of SFRC beams with high-strength reinforcement.

This paper focuses on the shear behavior of SFRC beams with high-strength reinforcement. The effect of steel fiber volume fraction and stirrup ratio on the shear behavior were investigated, including the load-deflection curve, concrete strain, stirrup strain, diagonal crack width, failure mode and shear capacity. The test results are mainly concerned with the effect of steel fiber on shear capacity, and the applicability of CECS38:2004 and ACI544.4R equations to calculate shear capacity of SFRC beam with high-strength reinforcement.

## 2. Experimental Program

### 2.1. Materials and Mixture Proportions

**Figure 1:**
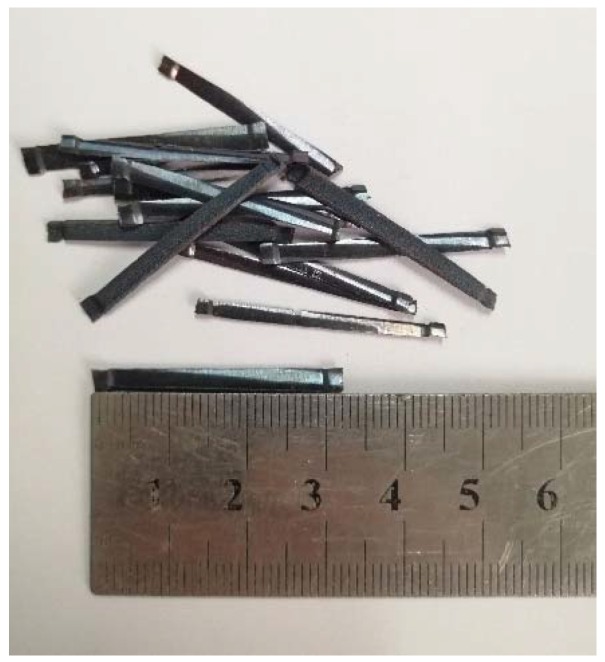
Mill-cut steel fiber.

The cement used in concrete was ordinary Portland cement (P.O 42.5) (Tianrui Group, Zhengzhou, China), coarse aggregate was gravel (Zhengzhou, China) with the largest diameter 20 mm, and fine aggregate was natural river sand (Zhengzhou, China). The steel fiber was mill-cut type (Harex Steel Fiber Co., Ltd., Shanghai, China), as shown in Figure 1 (fiber length of 32.2 mm, equivalent diameter of 0.92 mm, aspect ratio of 35, tensile strength of 700 MPa, density of 7.85 g/cm^3^). The mix proportions of SFRC were designed according to JTG472-2015 [34], as shown in Table 1. Three-cylinder test blocks (φ150 mm × 300 mm) and six prism test blocks (150 mm × 150 mm × 300 mm) were cast for each mix proportion. The cylinder test block was used to measure the splitting tensile strength, and the prism test block was used to measure the axial compression strength and elastic modulus. The reinforcement (Anyang Iron & Steel Group Co., Ltd., Anyang, China) used in the test were all high-strength reinforcement with diameters of 8 mm, 16 mm and 25 mm, respectively. The main parameters of high-strength reinforcement are shown in Table 2.

### 2.2. Details of Experimental Specimen

**Figure 2:**
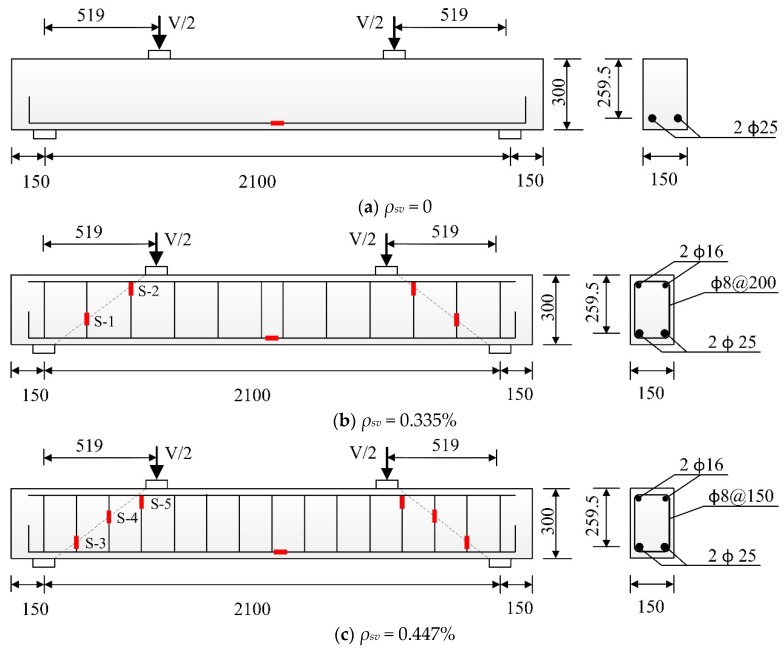
Dimension and reinforcement details of the experimental beams (Dimension in mm). (**a**) The specimens without stirrups; (**b**) The specimens with stirrup ratio 0.335%; (**c**) The specimen with stirrup ratio 0.447%.

According to CECS38:2004 [30], eight SFRC beams with high-strength reinforcement and three RC beams with high-strength reinforcement were produced. The length, width and height of all test beams were 2100 mm, 150 mm and 300 mm, respectively, and the shear span ratio were 2. The longitudinal bars were two steel bars with a diameter of 25 mm, the reinforcement ratio of longitudinal bars was 2.52%, and the concrete cover depths of all the beams were 20 mm. Two major variables considered in this study were the stirrup ratio (*ρ_sv_* = 0, 0.335%, 0.447%) and fiber volume fraction (*V_f_* = 0, 0.5%, 1%, 1.5%, 2%), the beams with the stirrup ratio of 0 or 0.335% contained five fiber volume fractions (*V_f_* = 0, 0.5%, 1%, 1.5%, 2%), and the dimension and reinforcement details are shown in Table 3 and Figure 2.

### 2.3. Test Procedure and Data Collection

**Figure 3:**
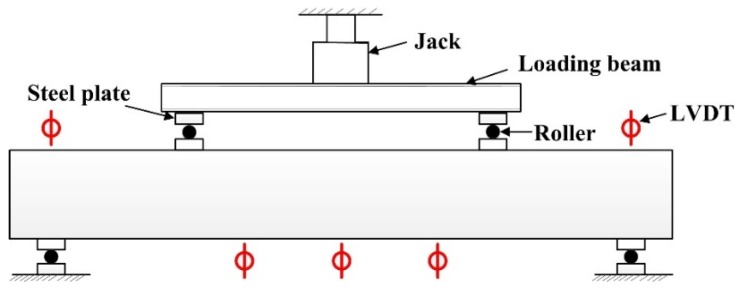
Loading diagram of experimental beam.

Figure 3 shows a schematic diagram of the loading of the test beam, and the test was carried out according to GB/T50152-2012 [35]. The maximum capacity of the jack (Hongshan Testing Machine Co., Ltd., Tianshui, China) in the test was 600 kN. 5 kN was a loading level before the diagonal section cracking, 15 kN was a loading level after the diagonal section cracking, and 5 kN was a loading level when the beam was close to failure. The number of the loading level for each beam was about ten, and all test beams were designed for shear failure. The sizes of the loading plates and supports were 250 mm (length) × 100 mm (width) × 40 mm (height).

**Figure 4:**
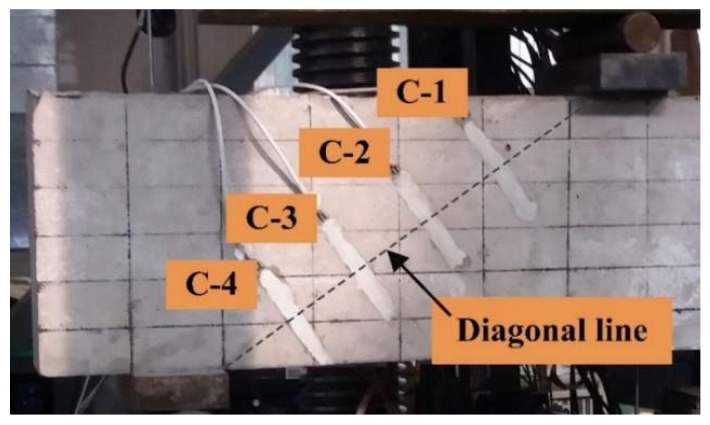
Positions of concrete strain gauges along the diagonal section.

As shown in Figure 3, LVDTs (Liyang city instrument and meter plant, Liyang, China) were arranged at the midspan, loading points and supports for measuring the vertical displacement there. The position of concrete strain gauges and reinforcement strain gauges (Jinli sensing element factory, Xingtai, China) are shown in Figure 2 and Figure 4, respectively. The white pieces orthogonal to the diagonal line in Figure 4 are concrete strain gauges, and the red short line in Figure 2 are reinforcement strain gauges, which were used to measure the tensile strain of the concrete and reinforcement. The first letter C and S of the strain gauge name represent concrete and steel bar, respectively.

## 3. Experimental Results and Data Analysis

### 3.1. Load-Deflection Curve

**Figure 5:**
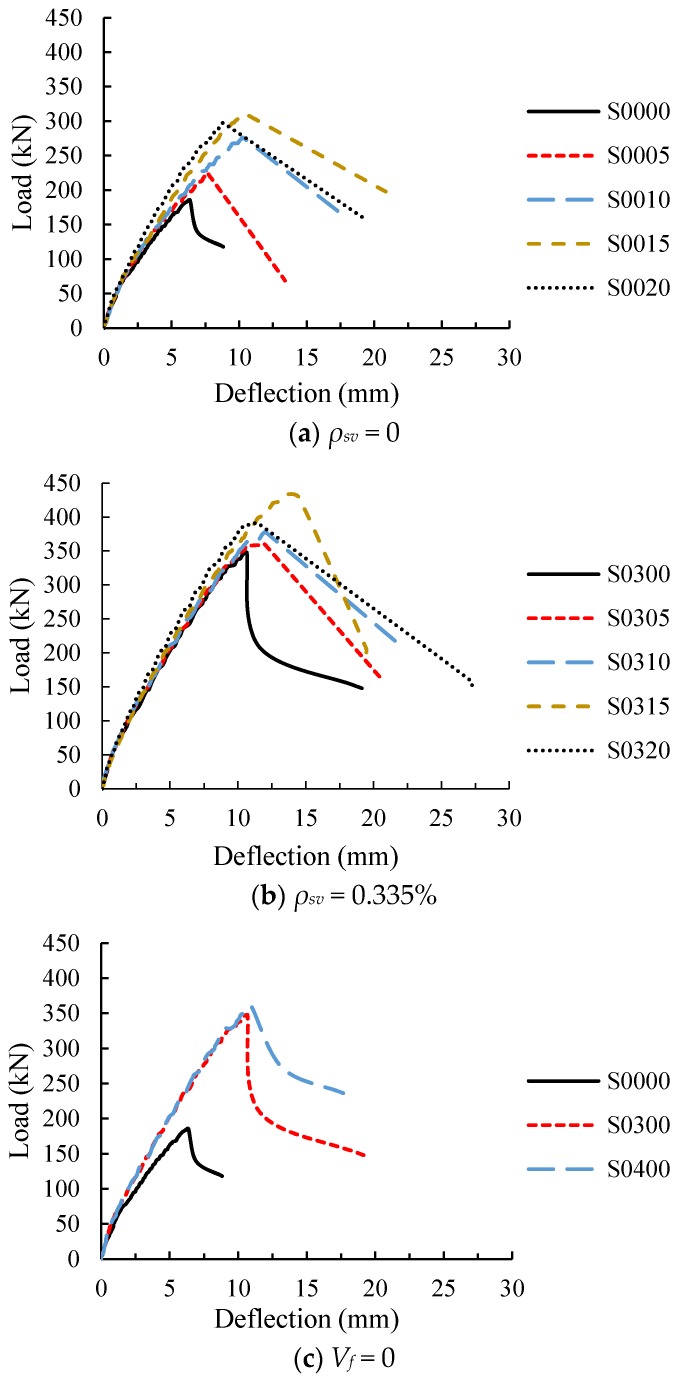
Load-deflection curves of specimens. (**a**) The test beams without stirrups; (**b**) The test beams with stirrup ratio 0.335%; (**c**) The test beams without steel fiber.

All the beams exhibited diagonal shear failure as designed, and the load-deflection curves obtained from the test are shown in Figure 5.

Figure 5a,b show the load-deflection curves of the beams with stirrups ratios of 0 and 0.335%, respectively. It can be seen that for the test beams without stirrups, the overall slope of the curve at ascending stage and the shear capacity were improved with increasing fiber volume fraction, indicating that steel fiber increases the stiffness and shear capacity of the beam. The reason for the increase in stiffness is that steel fiber increases the elastic modulus of concrete and the tensile stiffness. However, the effect of steel fiber on the elastic modulus is small (as shown in Table 3), and Ashour [36] points out that the stiffness of RC beam increases insignificantly with the increase of elastic modulus of concrete, because the height of concrete compressive zone decreases as the elastic modulus increases. Therefore, the influence of elastic modulus on stiffness can be ignored, and the stiffness of SFRC beam is higher than that of RC beam due to the effect of steel fiber in tensile zone. The result is also obtained by Yoo [18] and Meda [12] in their experimental studies. Similarly, for the beam with stirrups ratio 0.335%, steel fiber also increased the stiffness and shear capacity, but the effect of steel fiber on the beam with stirrups was less than that for the beam without stirrups, and it became significant only when the fiber volume fraction was not less than 1.5%. In addition, the steel fiber significantly increased the corresponding deflections when the beam without stirrups reached its ultimate load and failure. Although the steel fiber also increased both the deflections of ultimate load and failure for the beams with stirrups, the increments were less than 42%. The above results show that the steel fiber increases the stiffness of the test beams, as well as the deformation at failure, which means it reduces the brittleness of diagonal shear failure.

Figure 5c shows the load-deflection curves of RC beams with different stirrup ratio. It can be seen that the stirrups significantly increased the stiffness, ultimate load and damaged deflection of the test beam when the stirrup ratio increased from 0 to 0.335%. However, when the stirrup ratio continued to increase to 0.447%, the ultimate load increased by less than 4%.

In a word, both steel fiber and stirrups can improve the stiffness, ultimate load and damaged deflection of the beams, so RC beams with steel fiber or stirrups have a certain plastic deformation before the failure. The effect of steel fiber on the shearing mechanical properties of the beam without stirrups is obvious, but the stirrups reduce the improvement of the steel fiber. Therefore, it can be considered that the steel fiber can achieve the improvement of the stirrups on the shearing mechanical behavior of the beam.

### 3.2. Tensile Concrete Strain

**Figure 6:**
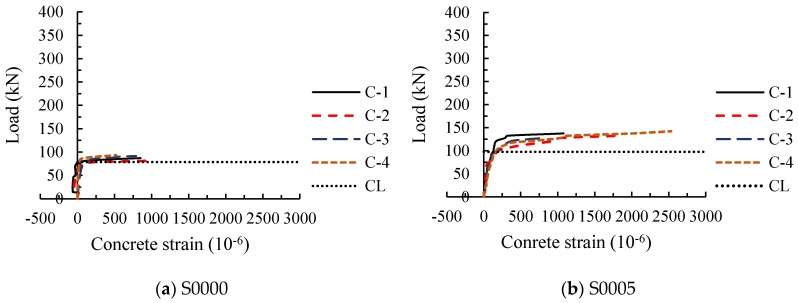

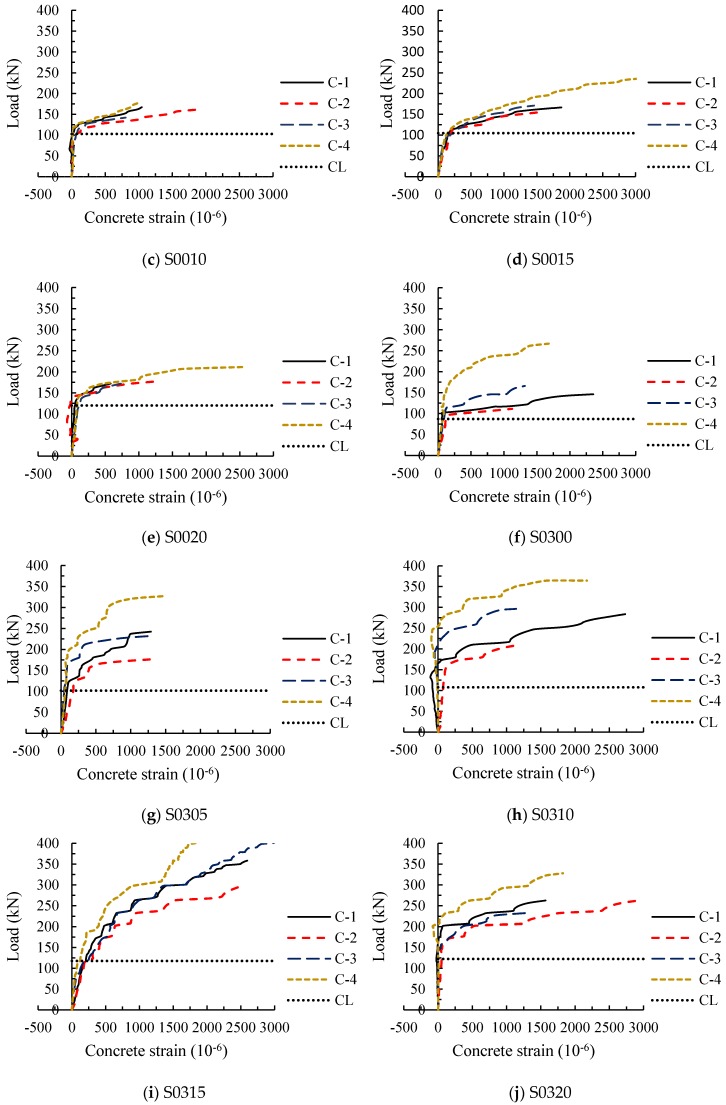

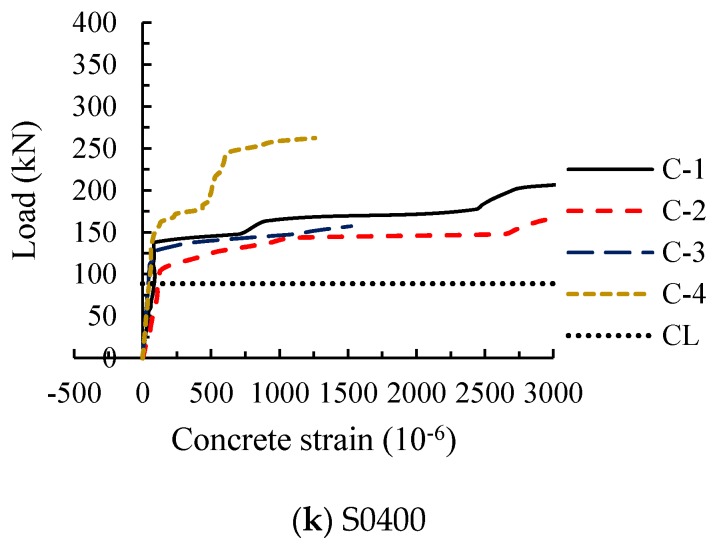
Load-concrete strain curves of specimens (CL: cracking load). (**a**) Beam S0000; (**b**) Beam S0005; (**c**) Beam S0010; (**d**) Beam S0015; (**e**) Beam S0020; (**f**) Beam S0300; (**g**) Beam S0305; (**h**) Beam S0310; (**i**) Beam S0315; (**j**) Beam S0320; (**k**) Beam S0400.

The load-concrete strain curves of 11 test beams obtained from four concrete strain gauges along the diagonal section (Figure 4) are shown in Figure 6.

In the test, it was observed that the diagonal crack at the web of the beam first appeared near the midpoint of the beam height. With the increase of load, the crack simultaneously extended to the directions of the loading point and support point. When a concrete strain increases faster than before, it means the crack extends to this measured point. If the difference between the cracking loads of the measured points is small, it indicates that the extension of the crack is rapid. And the increase speed of the concrete strain represents the development speed of the crack width.

Figure 6a shows the load-concrete strain curves of RC beam without stirrups. It can be seen from the diagram that after the cracking of the diagonal section, the four concrete strain gauges almost simultaneously increased rapidly and then failed to work. This indicates that as soon as the diagonal shear crack occurs at the web of the beam S0000, it extends very fast, and the crack width increased rapidly. After steel fiber was added in the beam, the cracking load of the diagonal section increased. And when the diagonal section of SFRC beams without stirrups cracked, the concrete strains at four measuring points almost rapidly increased at the same time (as shown in Figure 6b–d). However, compared with the beam S0000, the concrete strains underwent a gradual improvement with the increase of the load, and the degree of improvement varied with the steel fiber volume fraction, which were roughly 2 to 3 times that of the test beam S0000. It shows that steel fiber increases the diagonal cracking load, reduces the widening speed of the diagonal crack at the web of the beam, and increases the degree of concrete bearing in tension, but it is not effective to control the extension of the crack.

Load-concrete strain curves of RC beams with stirrups are shown in Figure 6f,k. Similar to the curves of SFRC beams without stirrups, the concrete strain increased gradually with the increase of load after it entered the rapid growth stage, but the concrete strains did not enter the rapid growth stage at the same time; that is, the cracking loads at four measuring points were obviously different. The results show that the addition of stirrups reduces the increase speed of concrete strain and increases the difference between the cracking loads of the measuring points, speculating that the stirrups effectively restrained the expansion and extension of the diagonal crack at the web.

For the test beams containing both stirrups and steel fiber, the corresponding loads were great when the four concrete strains entered the rapid growth stage, and the difference between the cracking loads was more obvious than that of the beams with stirrups and without steel fiber (Figure 6g,h,j). Besides, the growing degree of concrete strains were further enlarged. This indicates that the expansion and extension of the diagonal crack at the web are further restrained by the combined effect of steel fiber and stirrups.

### 3.3. Stirrup Strain

**Figure 7:**
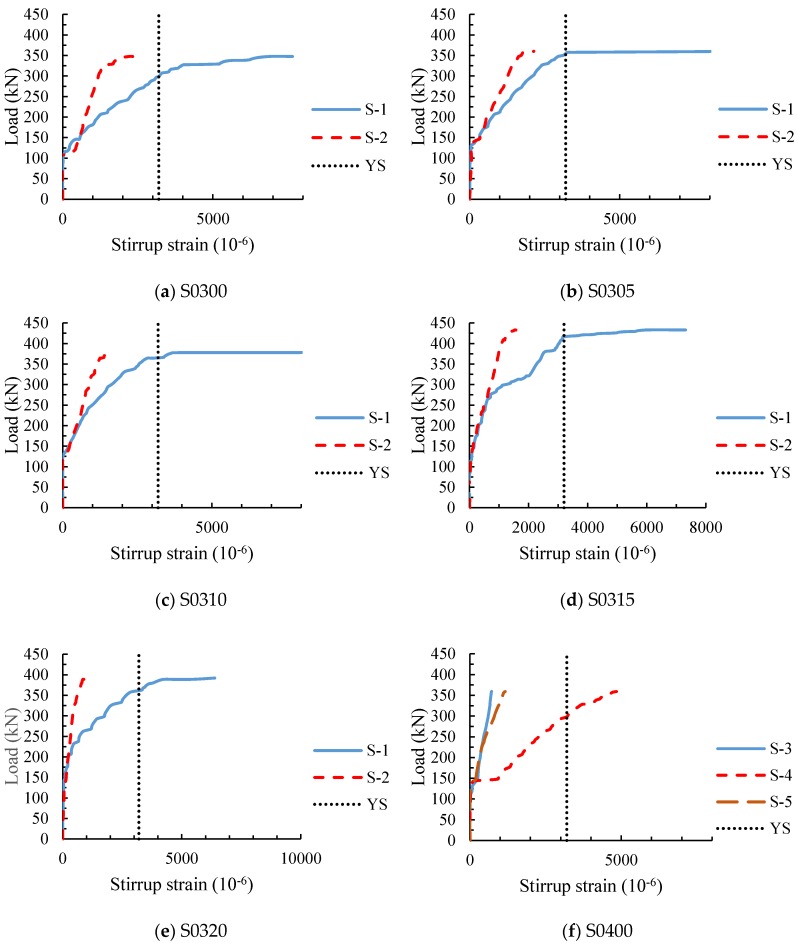
Load-stirrup strain curves of specimens (YS: yielding strain). (**a**) Beam S0300; (**b**) Beam S0305; (**c**) Beam S0310; (**d**) Beam S0315; (**e**) Beam S0320; (**f**) Beam S0400.

Figure 7 shows the load-stirrup strain curves of the test beams with stirrups, which was measured by the strain gauges attached to the stirrups along the diagonal section (Figure 2). From Figure 7a,f, it can be seen that the development of the stirrup strain is similar for the two test beams with different stirrup ratios. The stirrup strains were little before the initial diagonal cracking, and the tension was mainly resisted by the concrete at this stage. When the diagonal section cracked, the stirrup strains increased suddenly. This is mainly because the concrete around the stirrups was cracked and no longer bore the load, resulting in a sudden increase of the tension borne by the stirrups. As the load increased, the stirrup strains increased rapidly, especially the strain of the stirrup at the mid-point section of the shear span. After yielding, as the load increased slightly, the stirrup strains continued to increase rapidly. In contrast, for the beam with large stirrup ratio, the stirrup strain increased slowly, and the strain value was small when damaged.

For SFRC beams with stirrups, the measured load-stirrup strain curves are shown in Figure 7b–e. It can be seen that the development of the stirrup strains of SFRC beams are basically the same as that of RC beam S0300; the stirrup strains of the two measuring points were small before the cracking of diagonal section, and the strain S-1 increased rapidly as the load increased slightly after yielding. But there were differences between the curves of SFRC beams and beam S0300. Compared with RC beam S0300, the degree of slope reduction of the curves for SFRC beams was not as severe as that of RC beam S0300 when the diagonal section was cracking, and the larger the steel fiber volume fraction, the smaller the increase of the stirrup strain. This is because the steel fiber at the diagonal crack takes part of the tensile force and reduces the stirrup stress. After the diagonal section cracking, the strains of the beam S0300 at the two measured points were obviously different, while the strains of SFRC beams were still close at the early stage. This is caused by the fact that the steel fiber in concrete resists and transfers stresses, so that the stirrups at different sections of shear span are averagely stressed and resisted shear force together. Besides, the strain growths of S-2 for SFRC beams from yielding to failure were less than that of RC beam S0300. This is because that steel fiber reduces the increase of crack width at failure, resulting in the decrease of stirrup stress growth. 

### 3.4. Diagonal Crack Width

**Figure 8:**
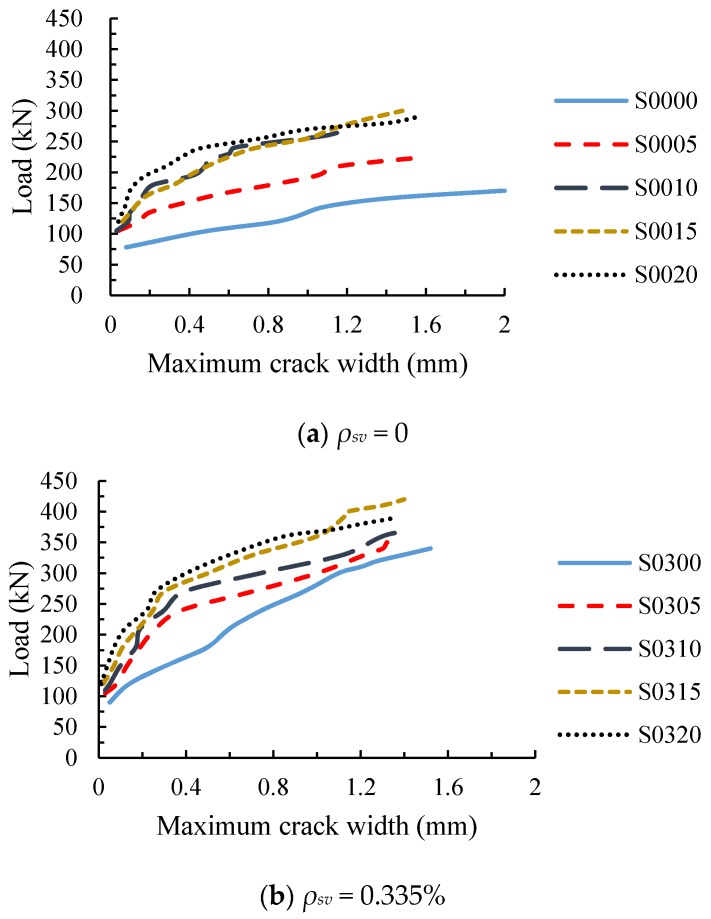

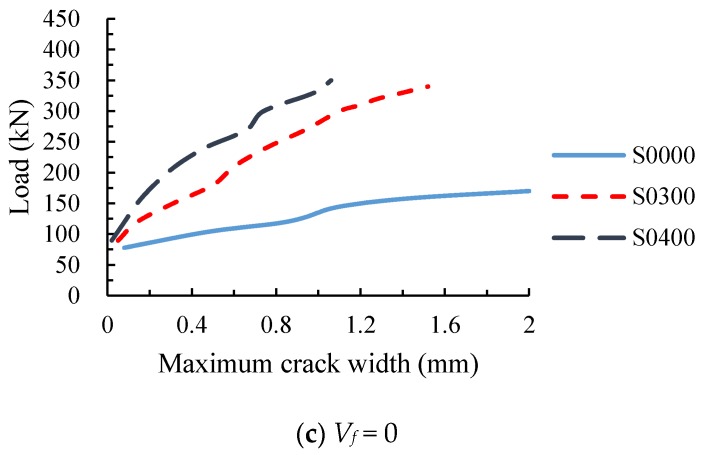
Load-maximum crack width of specimens. (**a**) The test beams without stirrups; (**b**) The test beams with stirrup ratio 0.335%; (**c**) The test beams without steel fiber.

The load-maximum diagonal crack width curves of the test beams are shown in Figure 8. Figure 8a shows the load-maximum diagonal crack width curves of the beams without stirrup. It can be seen from the diagram that the diagonal crack width of RC beam S0000 rapidly expanded to 0.08 mm after cracking, while the crack width of SFRC beams were not greater than 0.06 mm, indicating that steel fiber can reduce the width of the initial diagonal crack. Before the test beam S0000 was loaded to 140 kN, the width of the diagonal crack increased rapidly. After that, no new cracks were formed, and the width of the diagonal crack increased further. Compared with the beam S0000, the maximum width of the diagonal crack of SFRC beams under the same load were reduced due to the bonding and anchoring effect of the steel fiber at the crack.

In the early stage of diagonal crack development, the addition of steel fiber reduced the growth rate of the diagonal crack width, and the slopes of the load-maximum diagonal crack width curves decreased significantly. However, in the later stage, the diagonal crack width of SFRC beams grew faster than before, and the slopes of the load-maximum diagonal crack width curves were close to the beam S0000. In addition, when the fiber volume fraction of the beam increased from 0 to 1%, the effect of steel fiber on the diagonal crack width increased significantly, but it had less growth while the fiber volume fraction increased from 1% to 2%.

For RC beam with stirrup ratio of 0.335%, the steel fiber could also reduce the width of the diagonal crack, and the larger the steel fiber volume fraction was, the more the crack width decreased, as shown in Figure 8b. The diagonal crack width of SFRC beams with stirrups developed slowly at the beginning, and gradually became fast at the later stage. Due to the joint action of the stirrups and the steel fibers, the slow increase stages of the crack width for SFRC beams with stirrups were significantly longer, which were roughly 1.5 times that of SFRC beams without stirrups, and the slopes of the curves for the former were greater than that of the latter, that is, the diagonal crack width increased slower.

Figure 8c shows the load-maximum diagonal crack width curves of RC beams with different stirrup ratios. It can be seen from the diagram that the stirrups could also reduce the width of the diagonal crack, and the greater the stirrup ratio was, the more the crack width decreased. Compared with steel fiber, the stirrups played a greater role in controlling the development of the diagonal crack at the later stage, this is because the continuous stirrups are more effective in resisting high tensile stress than the discontinuous steel fiber.

### 3.5. Failure Modes

In the test, it was found that all the beams had a similar failure process, and the failure modes were not affected by the stirrup and steel fiber. The main process can be described as follows: when the applied load was 40–70 kN, small vertical cracks first appeared at the bottom of the beam between the loading points; as the load increased, more vertical cracks came out, and vertical cracks began to appear at the bottom of the shear span. When the load increased further, the vertical cracks in the shear span develop diagonally upward toward the loading points, forming flexure-shear cracks, and then the diagonal cracks were formed at the web of the beam in shear span. After this, the diagonal crack at the web continuously extended to the directions of loading point and support as the load increased. When the applied load was close to the ultimate load, the diagonal crack at the web of the beam extended to the vicinity of the loading point and support, and the width of the diagonal crack increased rapidly. Finally, the steel fiber was gradually pulled out until the concrete was crushed at the loading point, and the test beam was damaged.

**Figure 9:**
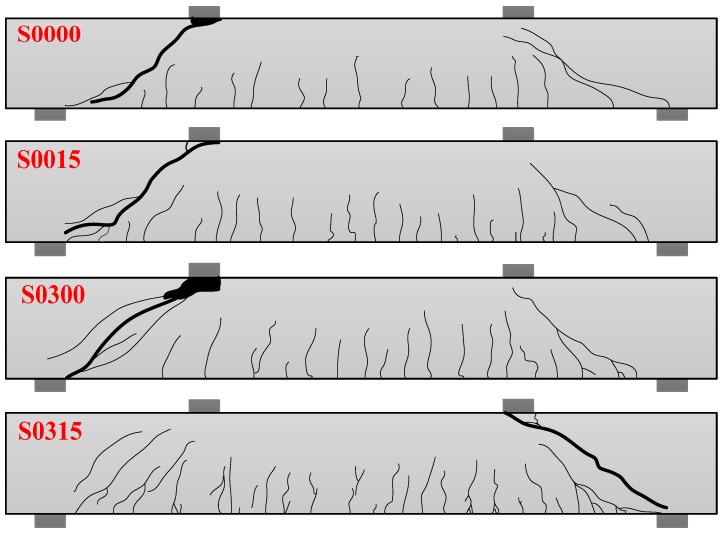
Failure modes.

The failure modes of four experimental beams are shown in Figure 9. It can be seen from the figure that the addition of steel fiber caused more vertical cracks and diagonal cracks in the test beam, and the crack spacing was reduced because the steel fiber transferred the higher stress at the crack section to the surrounding concrete matrix. In the process, more cracks are generated between existing cracks, or more branch cracks are generated. At the same time, steel fiber reduced the crack height, especially the height of vertical cracks between the loading points. In addition, the experimental phenomenon showed that, when the beam was damaged, the concrete of RC beams had obvious spalling at the loading point, but the deformation capacity of concrete in SFRC beams were improved by the bridging effect of steel fiber [37,38], which effectively prevented the concrete crushing and spalling at the compression zone.

## 4. The Analysis of Shear Capacity

### 4.1. Effect of Fiber Volume Fraction

**Figure 10:**
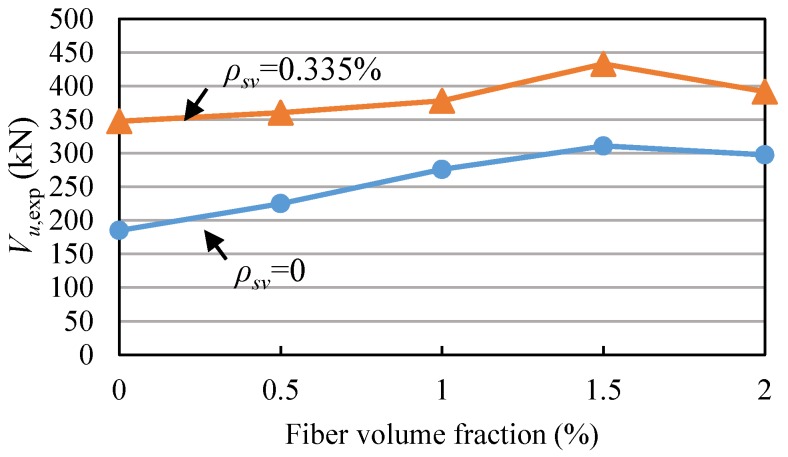
Relationship of shear capacity and fiber volume fraction.

The shear capacities of all the beams from the test are shown in Figure 10 and Table 3. It was found that when the stirrup ratio was the same, the shear capacities of the beams were improved by increasing the fiber volume fraction, except for the beams with a fiber volume fraction of 2%. The main reason is that when the fiber volume fraction is 2%, there are more defects in concrete because of too many steel fibers, and the tensile strength of SFRC (*V_f_* = 2%) is less than that of SFRC (*V_f_* = 1.5%). So the shear capacity after *V_f_* = 1.5% dropped. For the test beams without stirrups, the steel fiber significantly increased the shear capacity. When the steel fiber volume fraction was 0.5%, 1.0%, 1.5%, 2.0%, the shear capacity increased by 21.5%, 49.1%, 67.9%, and 60.7%, respectively. Compared to the beams without stirrups, the effect of steel fiber on the shear capacity of the beam with stirrups was smaller. When the steel fiber volume fraction was 0.5%, 1%, 1.5%, 2%, the shear capacity of the test beam was only increased by 3.7%, 8.7%, 24.6%, and 12.7%, respectively. The phenomenon that the shear capacities of the beams were improved by increasing the fiber volume fraction can be explained by the classical mechanical model provided by Swamy [39]. In addition, the results indicate that the effect of steel fiber on the shear capacity decreases with the increase of the stirrup ratio. This is due to the fact that the combined use of steel fiber and stirrups further restrains the crack development, it is not conducive to giving full play to the shear resistance of steel fiber.

### 4.2. Effect of Stirrup Ratio

**Figure 11:**
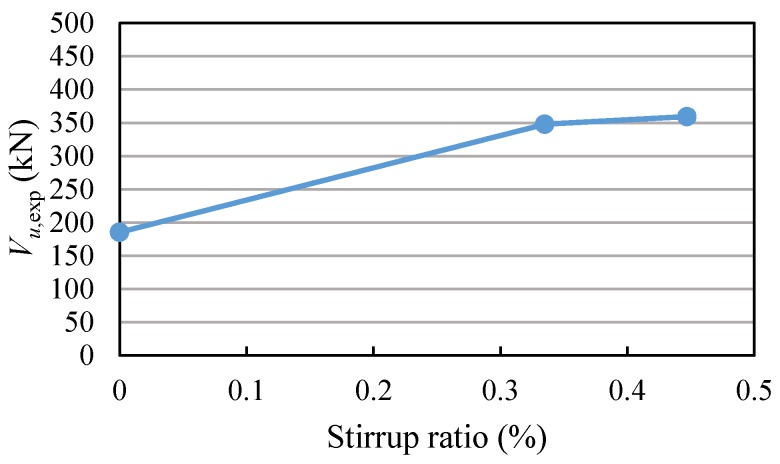
Relationship of shear capacity and stirrup ratio.

For RC beams with high-strength reinforcement, the larger the stirrup ratio, the greater the shear capacity, as shown in Figure 11. When the stirrup ratio increased from 0 to 0.335%, the shear capacity increased by 87.7%, indicating that the stirrups have a significant effect when the reinforcement ratio is relatively small. However, the shear capacity increased by only 3.3% while increasing the reinforcement ratio from 0.335% to 0.447%.

As shown in Figure 10, the addition of stirrups in SFRC beams could also increase the shear capacity, but it was not as significant as RC beam. When the fiber volume fraction of the beam was 0.5%, 1%, 1.5% and 2%, the shear capacity was improved by 60.2%, 36.9%, 39.3% and 31.6%, respectively. It shows that the effect of stirrup on shear capacity decreases as the steel fiber volume fraction increases. This is because the crack development of the beam with stirrups is further limited by the addition of steel fiber, so that the strains of stirrups are reduced and the effect of stirrups in resisting shear decreases. And the phenomenon can be explained by the mechanical model provided by Amin [15].

### 4.3. Comparison between the Measured Values and the Calculated Values of the Codes

#### 4.3.1. Comparison between the Measured Values and Calculated Values of CECS 38:2004

The Equation (1) in CECS38:2004 [30] is used to calculate the shear capacity of SFRC beam:

(1) $$V_{u}=V_{cf}+V_{s}=\frac{1.75}{\lambda +1}f_{t}bh_{0}\left(1+\beta_{v}\lambda_{f}\right)+f_{yv}\frac{A_{sv}}{s}h_{0}\;$$

where, *V_u_* is the shear capacity of the beam, *V_cf_* is the shear force provided by SFRC, *V_s_* is the shear force provided by the stirrup, *λ* is the shear span ratio, *f_t_* is the tensile strength of SFRC without considering the effect of steel fiber (obtained according to GB50010–2002), *b* is the section width, *h*_0_ is the effective depth of the section, *β_v_* is the influence coefficient of steel fiber (mill-cut profiled fiber 0.9, cut-off profiled fiber 0.6, shear-cut profiled fiber 0.5), *f_yv_* is the yield strength of the stirrups, *A_sv_* is the section area of the stirrups in the shear span, *s* is the stirrup spacing in the shear span, *λ_f_* = *V_f_* (*l_f_*/*d_f_*) is the characteristic value of fiber content, *V_f_* is the fiber volume fraction, *l_f_*/*d_f_* is the aspect ratio of fiber. Specifically, *V_cf_* is affected by the size effect, involved in the models of Spinella [40] and Dinh [41] about the shear strength of SFRC beam without stirrups. But based on the sufficient experimental results, it is stipulated by CECS38:2004 that the size effect can be ignored for the beam with stirrups, and the beam without stirrups whose height is less than 800 mm.

**Figure 12:**
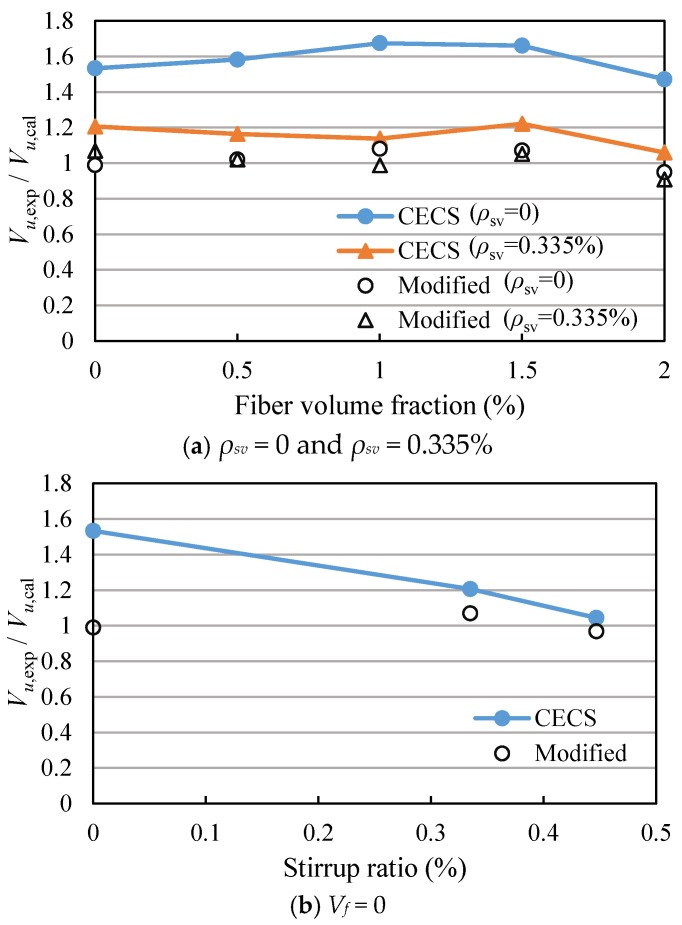
Comparison of test value and calculation value of CECS38:2004. (**a**) The test beams with stirrup ratio 0 and 0.335%; (**b**) The test beams without steel fiber.

The shear capacities of the beams calculated by Equation (1) are listed in Table 3. The ratios of the measured shear capacities to the calculated values of Equation (1) (*V_u_*_,exp_/*V_u_*_,CECS_) are between 1.04 and 1.67, as shown in Figure 12 and Table 3, indicating that the shear capacities of SFRC beams with high-strength reinforcement are not accurately predicted by Equation (1). Therefore, Equation (1) needs to be modified.

From Figure 12, it can be seen that the *V_u_*_,exp_/*V_u_*_,CECS_ of the beams without stirrups are all greater than 1.47, while the *V_u_*_,exp_/*V_u_*_,CECS_ of the beams with stirrups are close to 1. The results show that the shear force provided by SFRC decreases with the increase of the stirrup ratio. Therefore, it is necessary to introduce the influence coefficient of stirrup ratio into *V_cf_* of Equation (1), and Equation (1) can be expressed as

(2) $$V_{u}=\alpha V_{cf}+V_{s}=\alpha \frac{1.75}{\lambda +1}f_{t}bh_{0}\left(1+\beta_{v}\lambda_{f}\right)+f_{yv}\frac{A_{sv}}{s}h_{0}\;$$

*α* is the influence coefficient of stirrup ratio on *V_cf_*, and obtained by fitting the experimental results.

(3) $$\alpha =1.55-73\rho_{sv}\;$$

The Equations (2) and (3) are used to calculate the *V_u_*_,exp_/*V_u_*_,Modified_ of SFRC beams with high-strength reinforcement, and the results are showed in Figure 12 and Table 3. It can be seen that the shear capacity of SFRC beams with high-strength reinforcement are accurately predicted by the modified equation.

#### 4.3.2. Comparison between the Measured Values and Calculated Values of ACI544.4R

The Sharma’s equation [42] is adopted by ACI544.4R [31] for calculating the shear capacity of SFRC beam, expressed as

(4) $$V_{u}=V_{cf}+V_{s}=\frac{2}{3}f_{ts}{\left(\frac{h_{0}}{a}\right)}^{0.25}+f_{yv}\frac{A_{sv}}{s}h_{0}\;$$

where, *f_ts_* is the splitting tensile strength of the cylinder, and *a* is the shear span of the beam.

**Figure 13:**
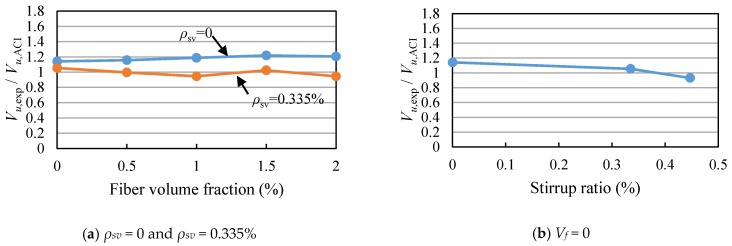
Comparison of test value and calculation value of ACI 544.4R. (**a**) The test beams with stirrup ratio 0 and 0.335%; (**b**) The test beams without steel fiber.

The ratios of the measured shear capacities to the calculated values of Equation (4) (*V_u_*_,exp_/*V_u_*_,ACI_) are between 0.93 and 1.20, as shown in Figure 13 and Table 3. It is concluded that the shear capacities of SFRC beams with high-strength reinforcement can be calculated according to the ACI544.4R. However, the Sharma’s equation is an empirical formula, and not established on an explicit mechanical model. Therefore, when the Equation (4) is applied to practical design, it should be based on enough experimental research.

## 5. Conclusions

In this paper, the shear behavior of SFRC beams with high-strength reinforcement was studied. The effect of steel fiber volume fraction and stirrup ratio on the shear behavior were investigated, including the load-deflection curve, concrete strain, stirrup strain, diagonal crack width, failure modes and shear capacity. Finally, the experimental values of the shear capacity were compared with the calculated values of CECS38:2004 and ACI544.4R.

The stress of the high-strength stirrup at service stage is higher than that of the conventional stirrup, and the diagonal crack width is related to the stress of the stirrup. Therefore, the next work is to further study the diagonal crack width of SFRC beams with high-strength reinforcement. The main research contents include verifying whether the diagonal crack width meets the requirements of the design codes at service stage and the calculated model of the diagonal crack width. Besides, the shear mechanism of the combined use of steel fiber and stirrups will be further studied.

Based on the above experimental results, the following conclusions are obtained:

(1) Steel fiber and stirrups can increase the stiffness, deflections at ultimate load and failure of RC beams, but the effect of the steel fiber decreases with the increase of the stirrup ratio.

(2) Steel fiber reduces the stirrup strain and the width of the diagonal crack due to the bridging effect at the crack. In addition, steel fiber increases the number of cracks, and reduces the crack height and crack spacing.

(3) Steel fiber increases the shear capacities of RC beams with high-strength reinforcement, but the increase range decreases with the increase of the stirrup ratio. Similarly, the stirrup also increases the shear capacities of the beams, but the increase effect of the stirrup on the shear capacity gradually decreases with the increase of the fiber volume fraction.

(4) The calculated values of the shear capacities of SFRC beams according to ACI544.4R are close to the experimental values, so it can be directly used to calculate the shear capacities of SFRC beams with high-strength reinforcement. The calculated values obtained from the formula in CECS38:2004 are obviously smaller than the experimental values and in good agreement with the test results after the influence coefficient of stirrup ratio is introduced into *V_cf_*.

(5) It is recommended that the maximum volume fraction of steel fiber added in RC beams is 1.5%, and the shear capacity of SFRC beam with high-strength reinforcement can be calculated by Equation (2) and (3).

## Figures and Tables

**Table 1:** Mix proportions.

Mix	W/B	Cement (kg)	Water (kg)	Coarse Aggregate (kg)	Fine Aggregate (kg)	Sand Ratio (%)	Steel Fiber (kg)	*V_f_* (%)
M-1	0.5	410	205	1150	735	39	0	0
M-2	0.5	410	205	1127	746	39	39.25	0.5
M-3	0.5	410	205	1104	756	39	78.5	1.0
M-4	0.5	410	205	1082	766	39	117.75	1.5
M-5	0.5	410	205	1058	777	39	157	2.0

Note: W/B is the water cement ratio and *V_f_* is the volume fraction of steel fiber.

**Table 2:** Major properties of high-strength reinforcement.

Diameter (mm)	Yield Strength (MPa)	Ultimate Tensile Strength (MPa)	Percent Elongation (%)
8	641.9	848.4	9.0
16	585.5	740.8	14.4
25	567.8	735.5	14.6

**Table 3:** Major details of the experimental beams.

Beam ID	Stirrup	*ρ_sv_* (%)	*V_f_* (%)	*f*_c_ (MPa)	*f*_ts_ (MPa)	*E_s_* (MPa)	*V_u_*_,exp_ (kN)	*V_u_*_,CECS_ (kN)	*V_u_*_,ACI_ (kN)	*V_u_*_,exp_/*V_u_*_,CECS_	*V_u_*_,exp_/*V_u_*_,ACI_	*V_u_*_,exp_/*V_u_*_,Modified_
S0000	-	0	0	33.03	3.72	23,713	185.20	120.80	162.35	1.53	1.14	0.99
S0005	-	0	0.5	34.45	4.46	24,856	225.07	142.24	194.65	1.58	1.16	1.02
S0010	-	0	1.0	36.08	5.33	25,968	276.12	164.88	232.61	1.67	1.19	1.08
S0015	-	0	1.5	37.13	5.85	27,211	310.90	187.24	255.31	1.66	1.22	1.07
S0020	-	0	2.0	35.26	5.66	26,802	297.61	202.23	247.02	1.47	1.20	0.95
S0300	φ8@200	0.335	0	33.03	3.72	23,713	347.61	288.20	329.76	1.21	1.05	1.07
S0305	φ8@200	0.335	0.5	34.45	4.46	24,856	360.45	309.65	362.05	1.16	0.99	1.02
S0310	φ8@200	0.335	1.0	36.08	5.33	25,968	378.02	332.29	400.02	1.14	0.95	0.99
S0315	φ8@200	0.335	1.5	37.13	5.85	27,211	433.13	354.64	422.71	1.22	1.02	1.05
S0320	φ8@200	0.335	2.0	35.26	5.66	26,802	391.79	369.64	414.72	1.06	0.95	0.91
S0400	φ8@150	0.447	0	33.03	3.72	23,713	359.10	344.01	385.56	1.04	0.93	0.97
Average										1.34	1.07	1.01
Standard deviation										0.23	0.11	0.05
Coefficient of variation										0.17	0.10	0.05

Note: The first letter of the beam ID represents the shear, the first two numbers represent the stirrup ratio, and the latter two number represent the fiber volume fraction. *f*_c_ is the compression strength of prism specimen at 28 days, *f*_ts_ is the splitting tensile strength of the cylinder specimen at 28 days, *E_c_* is the elastic modulus of concrete, *V_u_*_,exp_ is the shear capacity measured from the experiment, *V_u_*_,CECS_ is the shear capacity calculated by CECS38:2004, *V_u_*_,ACI_ is the shear capacity calculated according to ACI544.4R, and *V_u_*_,Modified_ is the shear capacity obtained by the modified equation of CECS38:2004.

## Notations:

*V_f_*	volume fraction of steel fiber
*l_f_*	length of steel fiber
*d_f_*	equivalent diameter of steel fiber
*λ_f_*	characteristic value of fiber content
*β_v_*	influence coefficient of steel fiber on shear capacity
*b*	width of beam section
*h*_0_	effective depth of beam section
*ρ_sv_*	stirrup ratio
*f*_c_	compression strength of prism specimen at 28 days
*f*_ts_	splitting tensile strength of cylinder specimen at 28 days
*f_t_*	tensile strength of SFRC without considering the effect of steel fiber
*E_c_*	elastic modulus of concrete
*f_yv_*	yield strength of stirrup
*A_sv_*	section area of stirrup in shear span
*s*	stirrup spacing
*λ*	shear span ratio
*V_u_*_,exp_	shear capacity measured from the experiment
*V_u_*_,CECS_	shear capacity calculated by CECS38:2004
*V_u_*_,ACI_	shear capacity calculated by ACI544.4R
*V_u_*_,Modified_	shear capacity calculated by the modified equation of CECS38:2004
*V_cf_*	shear force provided by SFRC
*V_s_*	shear force provided by the stirrup
*α*	influence coefficient of stirrup ratio on the *V_cf_*
